# Cell division cycle 7-kinase inhibitor PHA-767491 hydrochloride suppresses glioblastoma growth and invasiveness

**DOI:** 10.1186/s12935-016-0364-8

**Published:** 2016-11-18

**Authors:** Zubeyde Erbayraktar, Begum Alural, Resat Serhat Erbayraktar, Erdogan Pekcan Erkan

**Affiliations:** 1 Department of Biochemistry, Faculty of Medicine, Dokuz Eylul University, Izmir, Turkey; 2 Izmir Biomedicine and Genome Center, Dokuz Eylul University, Izmir, Turkey; 3 Department of Neuroscience, Institute of Health Sciences, Dokuz Eylul University, Izmir, Turkey; 4 Department of Neurosurgery, Faculty of Medicine, Dokuz Eylul University, Izmir, Turkey; 5 Pharmaplus Laboratories, Pharmaplus Ilac ve Saglik Urunleri Ltd. Sti, Izmir, Turkey

**Keywords:** Glioblastoma, Cell division cycle 7, CDC7 inhibitor, Cathepsin S, Kinase inhibitor

## Abstract

**Background:**

Genomic instability is a hallmark of cancer cells, and this cellular phenomenon can emerge as a result of replicative stress. It is possible to take advantage of replicative stress, and enhance it in a targeted way to fight cancer cells. One of such strategies involves targeting the cell division cycle 7-related protein kinase (CDC7), a protein with key roles in regulation of initiation of DNA replication. CDC7 overexpression is present in different cancers, and small molecule inhibitors of the CDC7 have well-documented anti-tumor effects. Here, we aimed to test the potential of CDC7 inhibition as a new strategy for glioblastoma treatment.

**Methods:**

PHA-767491 hydrochloride was used as the CDC7 inhibitor. Two glioblastoma cell lines (U87-MG and U251-MG) and a control cell line (3T3) were used to characterize the effects of CDC7 inhibition. The effect of CDC7 inhibition on cell viability, cell proliferation, apoptosis, migration, and invasion were analyzed. In addition, real-time PCR arrays were used to identify the differentially expressed genes in response to CDC7 inhibition.

**Results:**

Our results showed that CDC7 inhibition reduces glioblastoma cell viability, suppresses cell proliferation, and triggers apoptosis in glioblastoma cell lines. In addition, we determined that CDC7 inhibition also suppresses glioblastoma cell migration and invasion. To identify molecular targets of CDC7 inhibition, we used real-time PCR arrays, which showed dysregulation of several mRNAs and miRNAs.

**Conclusions:**

Taken together, our findings suggest that CDC7 inhibition is a promising strategy for treatment of glioblastoma.

**Electronic supplementary material:**

The online version of this article (doi:10.1186/s12935-016-0364-8) contains supplementary material, which is available to authorized users.

## Background

Glioblastoma is the most common malignant primary brain tumor. At the molecular level, glioblastoma is characterized by unique genomic alterations, which are used to define four molecular subtypes [1]. The existence of distinct tumor subtypes creates a structural complexity, together with inherent resistance, limits the efficacy of available treatment strategies. These findings highlight the unmet need to develop novel therapeutic strategies for glioblastoma.

Conventional treatment strategies aim to inhibit cell cycle progression to attenuate tumor growth. An alternative approach involves enhancing replicative stress in a targeted manner, which can be exploited for therapeutic purposes [2]. One of the direct ways to accomplish this goal is to target cell division cycle 7-related protein kinase (CDC7). CDC7 is a serine/threonine kinase that phosphorylates minichromosome maintenance protein 2 (MCM2) of the eukaryotic pre-replication complex [3], and this step is required for initiation of DNA replication [4, 5]. Moreover, CDC7 overexpression is present in different cancer types [6–10]. Based on these findings, targeting CDC7 kinase was suggested as a new approach for cancer therapy [11].

Small molecule inhibitors of CDC7 were shown to have anti-tumor activity in different cancer types [12–14]. PHA-767491 hydrochloride is one of the first-generation CDC7 inhibitors, and its anti-tumor activity is well characterized. Here, we report that CDC7 inhibition by PHA-767491 hydrochloride inhibits cell proliferation and induces apoptosis in glioblastoma cells. In addition, we found that CDC7 inhibition suppresses glioblastoma cell migration and invasion. By using real-time PCR arrays, we found that CDC7 inhibition alters the expression of several mRNAs and miRNAs in glioblastoma cells. In particular, CDC7 inhibition results in a significant decrease in cathepsin S mRNA and protein expression. Overall, our results indicate that CDC7 inhibition suppresses glioblastoma growth and invasiveness.

## Methods

### Cell culture

U87-MG, U251-MG, and 3T3 cells were obtained from American Tissue Culture Collection (ATCC). All cell lines were cultured in Dulbecco’s Modified Eagle’s Medium (DMEM) supplemented with 10% fetal bovine serum (FBS), 2% l-glutamine, and 1% penicillin–streptomycin. Cells were maintained at 37 °C and in 5% CO_2_ atmosphere.

### CDC7 inhibitor

PHA-767491 hydrochloride (Sigma-Aldrich, Germany) was used as the CDC7 inhibitor. Lyophilized PHA-767491 hydrochloride was dissolved in nuclease-free water to achieve a final concentration of 5 mM, and aliquoted in 100 µl volume. Aliquots were stored at −20 °C, and a fresh aliquot was used for each experiment.

### Cell viability assay

5×10^3^ U87-MG and U251-MG cells were seeded in a 96-well plate 24 h before treatment. Next day, cells were treated with inhibitor (10 µM final concentration), solvent control (water), or left untreated. Seventy-two hours after treatment, 10 µl of PrestoBlue cell viability reagent (Invitrogen, Thermo Fisher Scientific, USA) was added onto the cells to assess cell viability. Relative cell viability was calculated by setting the viability of solvent control as 100%. Experiments were repeated at least three times.

### Cell proliferation assay

For synchronization, U87-MG and U251-MG cells were maintained in culture medium supplemented with 1% FBS for 24 h. Then, 1 × 10^4^ U87-MG and U251-MG cells were seeded in a 96-well plate. Next day, cells were treated with inhibitor (2.5 or 10 µM final concentration), solvent control (water), or left untreated. Seventy-two hours after treatment, bromodeoxyuridine (BrdU) cell proliferation ELISA kit (Cell Signaling, #5492) was used according to the manufacturer’s instructions. Rate of proliferation in cells treated with solvent control was set as 100% to calculate relative cell proliferation rate.

### Detection of cell death

U87-MG and U251-MG cells (5 × 10^3^ cells/well) were seeded in a 96-well plate. Next day, cells were treated with inhibitor [(2.5 or 10 µM final concentration), solvent control (water), or left untreated. Twenty-four hours after treatment, cells were lysed, and cell death detection ELISA^Plus^ kit (Roche, #11774425001) was used according to the manufacturer’s instructions. Relative level of DNA fragmentation (indicator of apoptosis) was calculated according to the following formula:

$${\text{Relative DNA fragmentation }} = \, {{{\text{Absorbance}}_{{[{\text{CDC7i}}]}} } \mathord{\left/ {\vphantom {{{\text{Absorbance}}_{{[{\text{CDC7i}}]}} } {{\text{Absorbance}}_{{[{\text{solvent control}}]}} }}} \right. \kern-0pt} {{\text{Absorbance}}_{{[{\text{solvent control}}]}} }}$$

### Immunoblotting

To analyze total MCM2 and p-MCM2 (S40 + S41) protein levels, U87-MG and U251-MG cells were treated with PHA-767491 hydrochloride (10 µM final concentration) for 12 h. To analyze CTSS protein expression, U87-MG and U251-MG cells were treated with CDC7 inhibitor (2.5 µM final concentration), and cells were harvested at three different time points (24, 48, and 72 h). Afterwards, cells were lysed in a cocktail (RIPA buffer supplemented with protease and phosphatase inhibitors). Equal amount (50 µg) of protein samples were separated on 4–10% Tris–Cl polyacrylamide gel, and proteins were transferred to polyvinylidene difluoride (PVDF) membranes. The following antibodies were used in Western blotting experiments: p-MCM2 (S40/S41) antibody (1:2000, abcam, ab70371); MCM2 antibody (1:1000, abcam, ab108935); cathepsin-S (1:1000, abcam, ab134157) and beta-actin antibody (1:10,000, abcam, ab8227). After incubation with primary antibodies, membranes were subsequently incubated with HRP-labeled goat-anti-rabbit immunoglobulins (Santa Cruz). SuperSignal West Pico Chemiluminescent Substrate (Thermo Scientific) was used for protein detection.

### RNA isolation

miRNeasy kit (#217004; Qiagen GmbH, Hilden, Germany) was used according to the manufacturer’s protocol to isolate total RNA from glioblastoma cells. RNA samples were eluted in nuclease-free water, and stored at −80 °C until further analysis.

### Reverse-transcription

RevertAid First Strand cDNA Synthesis Kit (#K1622; Thermo Scientific Fisher) was used to reverse-transcribe RNA samples for mRNA expression analysis. miScript RT II kit (Qiagen GmbH, Hilden, Germany) was used to reverse-transcribe RNA samples for miRNA expression analysis. Equal amount (1 μg) of RNA samples were used for reverse transcription. cDNA samples were diluted with nuclease-free water, and stored at −20 °C until analysis.

### Real-time PCR

All real-time PCR experiments were performed on a LightCycler 480 instrument (Roche). In each experiment, three technical replicates per sample and a no-template control (NTC) were used. The following primer sequences were used: CTSS-F: GCCTGATTCTGTGGACTGG, CTSS-R: GATGTACTGGAAAGCCGTTGT; GAPDH-F: GCAAATTCCATGGCACCGT; GAPDH-R: TCGCCCCACTTGATTTTGG. GAPDH was used as a housekeeping gene to normalize mRNA expression. Relative mRNA expression was calculated by using the formula 2^−ddCt^ [15].

### Migration assay

U87-MG and U251-MG cells were seeded at a density of 2.5  ×  10^5^ cells/well in 6-well plates, and incubated in DMEM supplemented with 10% FBS for 24 h. A confluent monolayer of each well was scratched with a 200 μl pipette tip, and cells were washed twice with 1× PBS. Then, fresh growth medium containing 10% FBS, with or without CDC7 inhibitor (final concentration: 2.5 or 10 µM) was added onto the cells. For each well, the wound area was marked, and images were acquired immediately after the scratch (0 h), and at 48 h on a phase-contrast inverted microscope (Olympus, CKX41; magnification: 10×). At each time point, the same wounded area was selected for image acquisition. The number of migrated cells was counted on ImageJ v.1.42 software as described previously [16]. All experiments were performed in triplicate, and each experiment was repeated at least three times.

### Invasion assay

Cell invasion experiments were performed using 24-well invasion chambers (pore size: 8 µm, 24 well; Greiner) coated with 250 µg/ml Matrigel (#356234; BD Bioscience). U87-MG and U251-MG cells (2 × 10^4^/well) were plated in medium without FBS, and 10% FBS in the bottom chamber served as chemoattractant. Twenty-four hours after seeding, cells were stained with Diff-Quick Staining Set (#NC0674866; Siemens) and cells that did not cross the transfilter were removed with cotton swabs. Invaded cells were visualized by phase-contrast microscopy, and counted on Image J v.1.42 software.

### Real-time PCR arrays

Human Cancer Drug Targets PCR Array (PAHS-507ZF-12; Qiagen GmbH, Hilden, Germany) and Brain Cancer miRNA PCR Array (MIHS-108Z; Qiagen GmbH, Hilden, Germany) were used to analyze the changes in cellular mRNA and miRNA expression profiles upon CDC7 inhibitor treatment. Twenty-four hours before treatment, 1 × 10^5^ U87-MG cells were seeded in 6-well plates. Next day, cells were treated with inhibitor (final concentration: 2.5 µM) or solvent control (water). Total RNA isolation was performed as described previously. miScript RT II kit (Qiagen GmbH, Hilden, Germany) and RT2 First Strandt kit (Qiagen GmbH, Hilden, Germany) were used for reverse-transcription reactions. Input RNA amount was 400 ng for Human Cancer Drug Targets PCR array, and 500 ng for Brain Cancer miRNA PCR Array.

Three biological replicates were used for each real-time PCR experiment. All real-time PCR experiments were performed on a LightCycler 480 instrument (Roche). A cut-off value of 1.5-fold was used to identify the significant hits.

### Statistical analysis

All statistical analyses were performed on GraphPad Prism v.6.0 for Mac OS X (GraphPad Software, La Jolla, California, USA). Independent samples *t* test was used to analyze the differences between groups. P < 0.05 were considered as statistically significant.

## Results

### CDC7 inhibition decreases glioblastoma cell viability in a time- and dose-dependent fashion

**Fig. 1:**
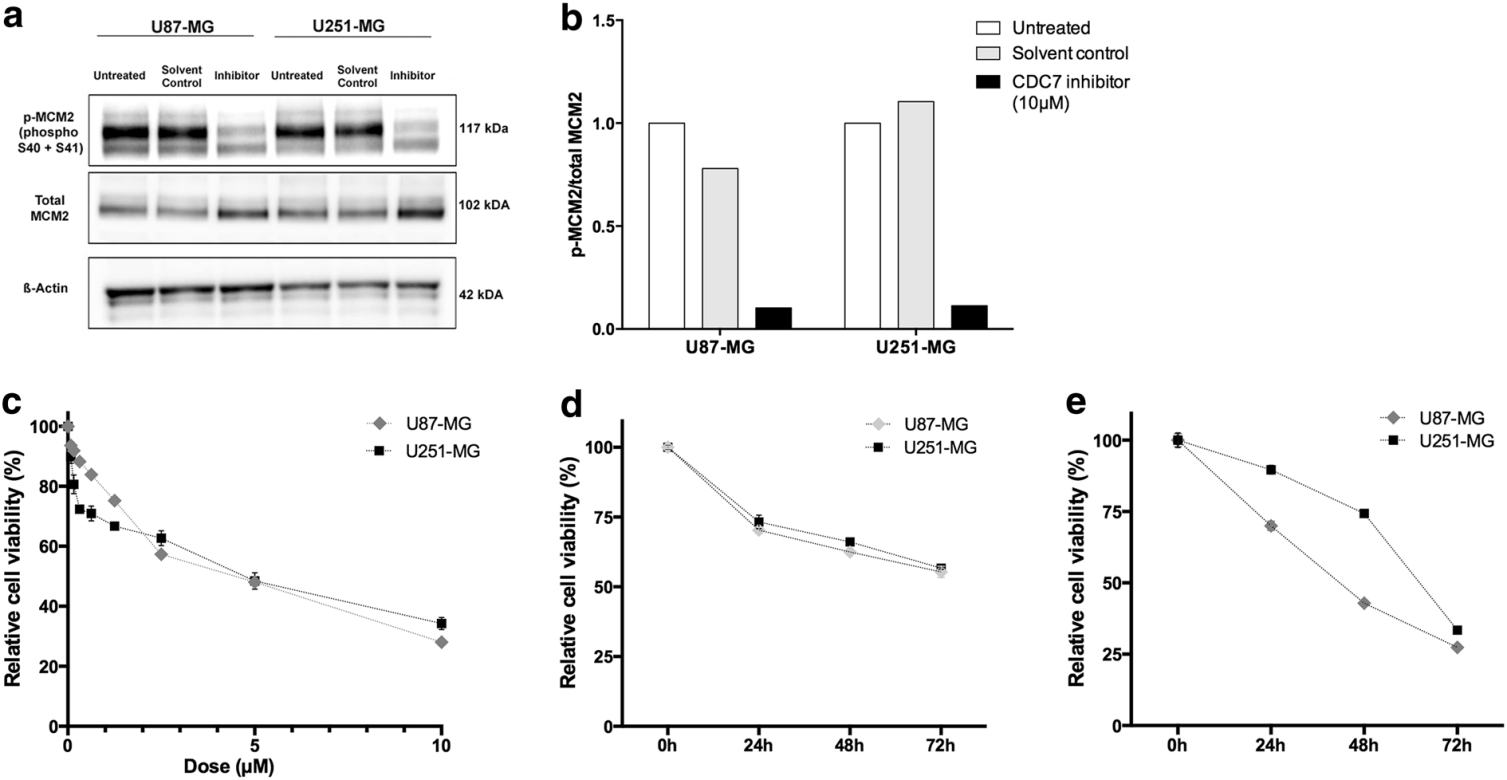
CDC7 inhibition decreases glioblastoma cell viability in a time- and dose-dependent fashion. **a** Protein levels of total MCM2 and p-MCM2 (S40 + S41) were analyzed with immunoblotting to confirm pharmacodynamic efficacy of CDC7 inhibition. Treatment with CDC7 inhibitor (10 μM) leads to a significant reduction in p-MCM2 (S40 + S41) expression in U87-MG and U251-MG cell lines. **b** ImageJ software was used to quantify the signal intensities in immunoblots. **c** U87-MG and U251-MG cells were treated with different concentrations of CDC7 inhibitor (0–10 μM) for 72 h to determine the IC50 value. **d** U87-MG and U251-MG cells were treated with CDC7 inhibitor (2.5 μM) for 24, 48, and 72 h, and PrestoBlue cell viability reagent (Thermo Fisher Scientific, #A13261) was used to assess cell viability. **e** Under similar experimental conditions, U87-MG and U251-MG cells were treated with CDC7 inhibitor (10 μM) for 24, 48, and 72 h, and cell viability was assessed as described previously. Data represent mean ±SEM. of three independent experiments. [*P < 0.05, **P < 0.01 and ***P < 0.001 for treated cells vs control]

Inhibition of MCM2 phosphorylation at CDC7-dependent site Ser40/41 is a pharmacodynamic parameter of CDC7 inhibition [12]. To confirm this finding, we treated U87-MG and U251-MG cells with PHA-767491 hydrochloride (10 µM final concentration) for 12 h, and analyzed total MCM2 and phospho-MCM2 (S40 + S41) protein expression. Our results indicate that PHA-767491 hydrochloride treatment leads to significant reduction in p-MCM2 (S40 + S41) expression both cell lines (Fig. 1a, b).

Next, we aimed to determine the half maximal inhibitor concentration (IC_50_) of PHA-767491 hydrochloride. To do this, we treated U87-MG and U251-MG cells with different concentrations of PHA-767491 (0–10 µM) for 72 h, and analyzed cell viability. For both cell lines, the IC_50_ concentration was approximately 2.5 µM (Fig. 1c).

After determining the IC_50_ value, we aimed to analyze how glioblastoma cell viability changes in response to CDC7 inhibition. We treated U87-MG and U251-MG cells with different concentrations of CDC7 inhibitor (2.5 and 10 µM final concentration), and determined that treatment with 2.5 µM PHA-767491 hydrochloride decreased cell viability by approximately 45% in both cell lines (Fig. 1d). Similarly, treatment with 10 µM PHA-767491 hydrochloride decreased cell viability by approximately 75% in U87-MG cells, and 70% in U251-MG cells (Fig. 1e).

To explore the effects of CDC7 inhibition on non-tumorigenic cells, we used non-transformed 3T3 cells as control cell line. Treatment with PHA-767491 hydrochloride resulted in a modest decrease in cell viability (Additional file 1: Fig. S1a). On the other hand, we determined significant decrease in cell proliferation (Additional file 1: Fig. S1b). Contrary to glioblastoma cells, CDC7 inhibition did not cause a significant increase in the level of DNA fragmentation in 3T3 cells (Additional file 1: Fig. S1c). Overall, these findings indicate that PHA-767491 hydrochloride effectively decreases cell viability in glioblastoma cells in a time-dependent fashion, and CDC7 inhibition exerts limited effects on non-tumorigenic cells.

### CDC7 inhibition inhibits glioblastoma cell proliferation, and induces apoptosis

**Fig. 2:**
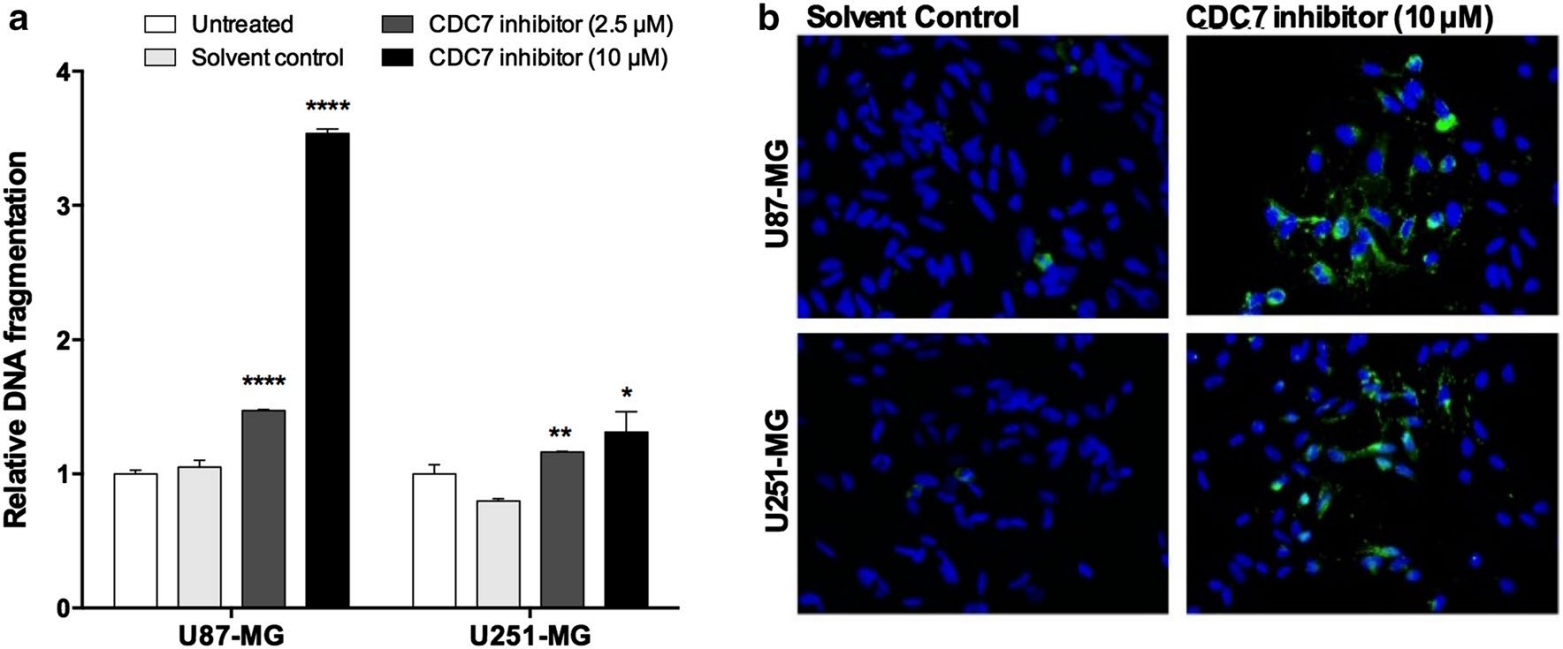
CDC7 inhibition induces apoptosis in glioblastoma cells. U87-MG and U251-MG cells were treated with different concentrations of CDC7 inhibitor (2.5 and 10 µM) for 24 h. Then, **a** Cell Death Detection ELISA^Plus^ kit (Roche, #11544675001) and **b** Annexin-V staining were used to analyze apoptotic cell death. Data represent mean ± SEM of three independent experiments. [*P < 0.05, **P < 0.01 and ***P < 0.001 for treated cells vs control]

PHA-767491 hydrochloride is able to induce apoptotic cell death [12], independent of p53 status of tumor cells. Our next question was to determine whether CDC7 inhibition would also induce apoptosis in glioblastoma cells. We found that CDC7 inhibitor treatment for 24 h results in a significant increase in DNA fragmentation in both U87-MG cells (3.54-fold compared to control) and U251-MG cells (1.31-fold compared to control) (Fig. 2a). Under similar experimental conditions, we performed Annexin V staining, which also confirmed that CDC7 inhibition induces apoptosis in both cell lines (Fig. 2b).

**Fig. 3:**
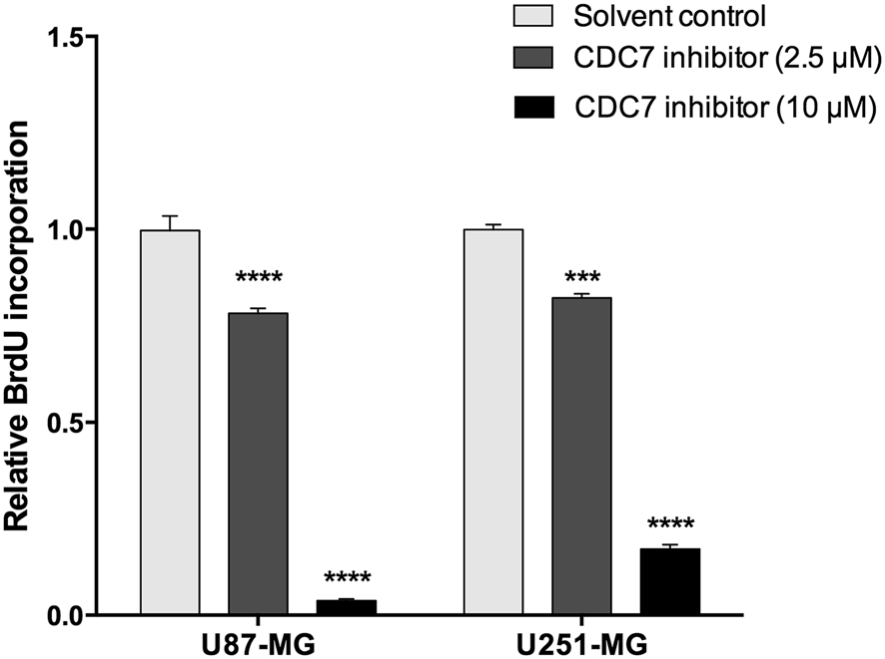
CDC7 inhibition suppresses glioblastoma cell proliferation. U87-MG and U251-MG cells were treated with different concentrations of CDC7 inhibitor (2.5 and 10 μM) for 72 h. Then, a chemiluminescent BrdU incorporation assay (Cell Signaling, #5492) was used to analyze the rate of cell proliferation. Data represent mean ± SEM of two independent experiments. [*P < 0.05, **P < 0.01, ***P < 0.001 and ****P < 0.0001 for treated cells vs control]

Another consequence of CDC7 inhibition is suppression of cell proliferation, which is demonstrated in multiple cell lines [12]. To determine if CDC7 inhibition also suppresses cell proliferation in glioblastoma cells, we used a chemiluminescent bromodeoxyuridine (BrdU) incorporation assay. Treatment with 2.5 µM of PHA-767491 hydrochloride resulted in approximately 20% decrease in cell proliferation in both cell lines (Fig. 3). Similarly, treatment with 10 µM of PHA-767491 hydrochloride resulted in 96% decrease in cell proliferation in U87-MG cells, and 83% decrease in cell proliferation in U251-MG cells (Fig. 3). Taken together, these results demonstrate that CDC7 inhibition suppresses glioblastoma cell proliferation, and induces apoptosis.

### CDC7 inhibition suppresses migration and invasion of glioblastoma cells

**Fig. 4:**
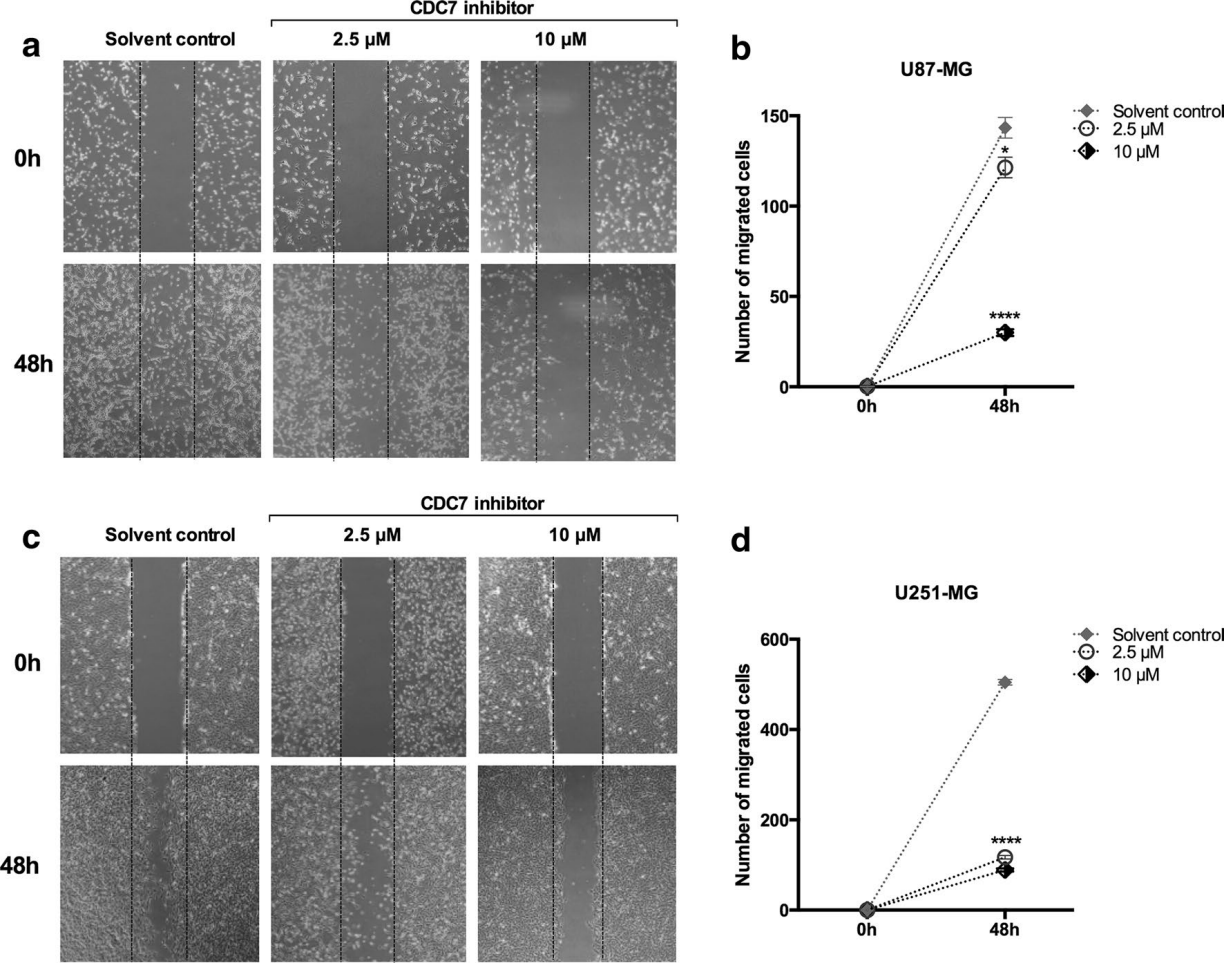
CDC7 inhibition suppresses glioblastoma cell migration. Cells were scratched with a pipette tip, and then incubated with CDC7 inhibitor (10 μM) for 0–72 h. Migrating cells were visualized under a phase contrast microscope. **a** Representative images of migrated U87-MG and **c** U251-MG cells. **b**, **d** Image J-Cell counter plugin (https://imagej.nih.gov/ij/plugins/cell-counter.html) was used to quantify the number of migrated cells. Data represent mean ± SEM of three independent experiments. [*P < 0.05, **P < 0.01, ***P < 0.001 and ****P < 0.0001 for treated cells vs control]

A common feature of glioblastoma cells is their aggressive phenotype, which is characterized by their migration and invasion abilities. Our next goal was to identify whether CDC7 inhibition has any effect on glioblastoma cell migration and invasion. Using a wound-healing assay, we found that CDC7 inhibitor treatment caused a significant decrease in the number of migrated cells in both cell lines (Fig. 4). At maximal dose (10 µM), we determined that the migration ability of both cell lines was diminished significantly (Fig. 4b, d). At IC_50_ dose, the degree of suppression was more prominent in U251-MG cells, compared to U87-MG cells.

**Fig. 5:**
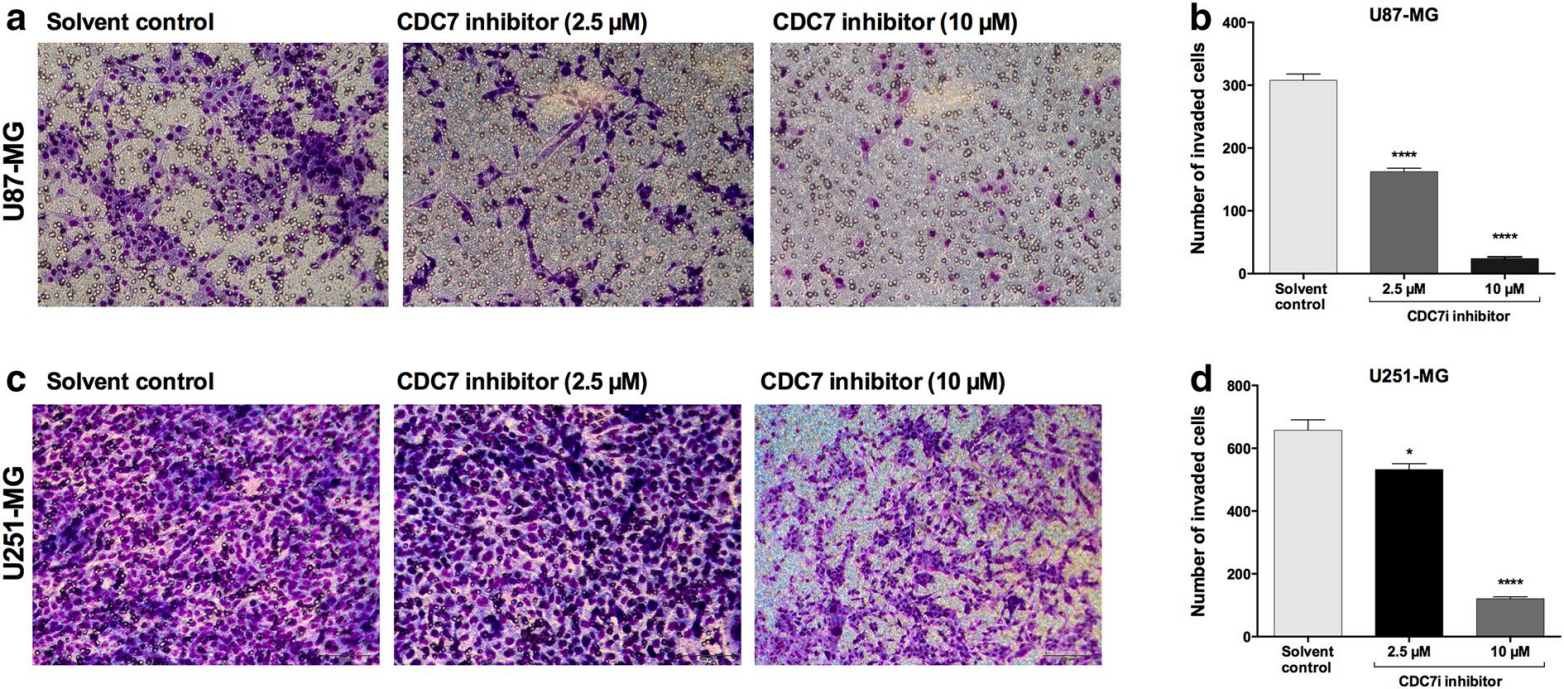
CDC7 inhibition suppresses glioblastoma cell invasion. **a** Representative images of invaded U87-MG and **c** U251-MG cells after 24-h culture in Matrigel invasion chambers. **b**, **d** ImageJ-Cell counter plugin (https://imagej.nih.gov/ij/plugins/cell-counter.html) was used to quantify the number of invaded cells. The invasiveness of U87-MG and U251-MG cells was attenuated with the CDC7 inhibitor treatment. Data represent mean ± SEM of three independent experiments. [*P < 0.05, **P < 0.01 ***P < 0.001 and ****P < 0.0001 for treated cells vs control]

In parallel, we also performed a transwell invasion assay to test the effect of CDC7 inhibition on glioblastoma cell invasion. Treatment with CDC7 inhibitor at maximal dose (10 µM) caused a significant decrease in number of invaded cells in both U87-MG cells (Fig. 5a, b) and U251-MG cells (Fig. 5c, d). However, at IC_50_ dose, the degree of inhibition was considerably less in U251-MG cells, compared to U87-MG cells. Taken together, these results indicate that CDC7 inhibition suppresses glioblastoma cell migration and invasion.

### Molecular consequences of CDC7 inhibition at the transcriptomic level

To understand the molecular consequences of CDC7 inhibition, we used commercial real-time PCR arrays to analyze the changes in expression levels of several mRNAs and miRNAs.

**Fig. 6:**
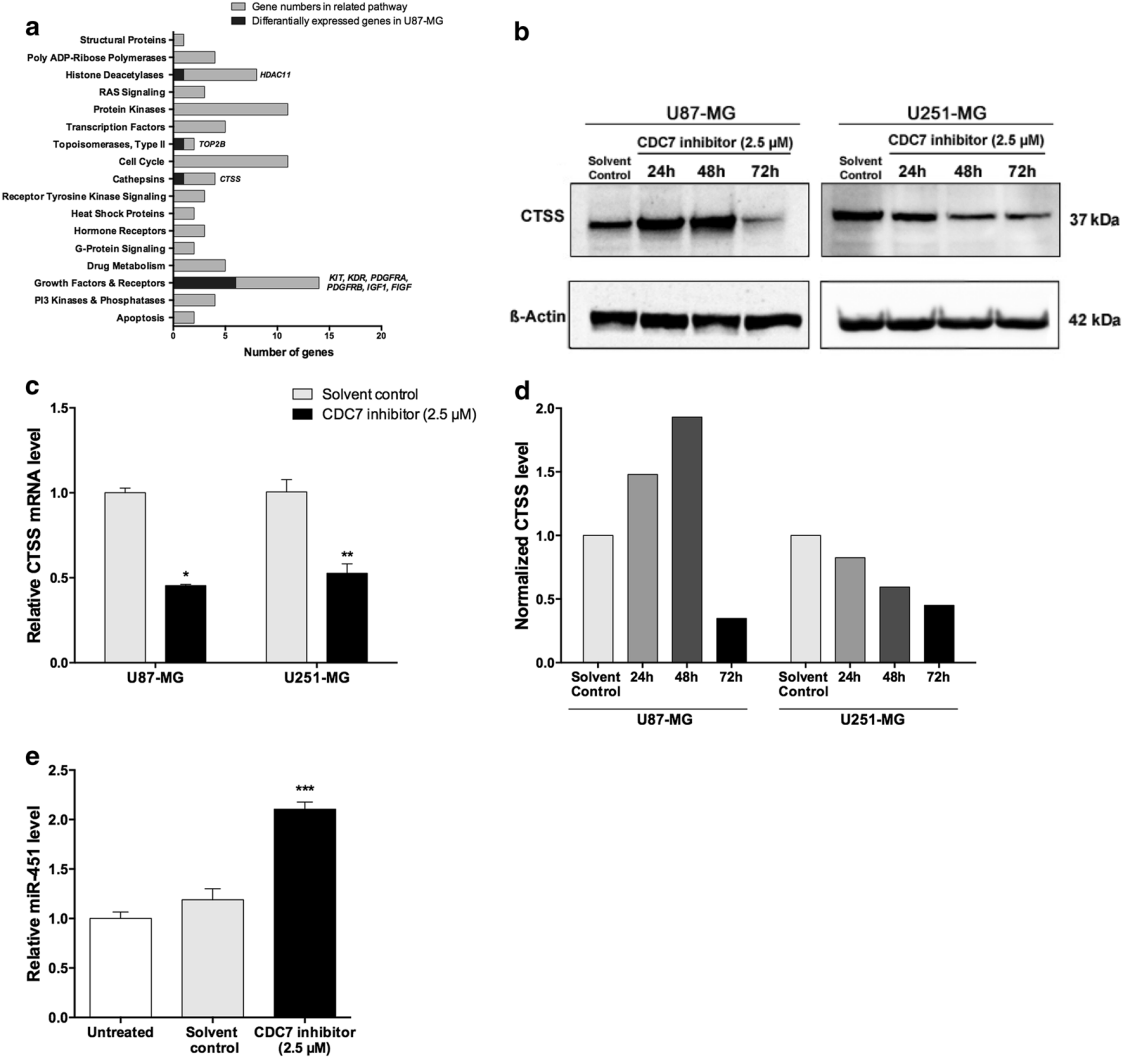
Dysregulated mRNAs and miRNAs in response to CDC7 inhibition. Commercial real-time PCR arrays were used to analyze differential mRNA and miRNA expression profiles in response to CDC7 inhibition. **a** List of cellular pathways analyzed by real-time PCR array. Differentially expressed mRNAs are indicated in italic. **b**, **d** Immunoblot analysis of CTSS in glioblastoma cell lines in response to CDC7 inhibition, and its quantification. **c** Real-time PCR analysis of CTSS mRNA expression levels in glioblastoma cell lines in response to CDC7 inhibition. **e** Analysis of miR-451 expression in U87-MG cell line in response to CDC7 inhibition [*P < 0.05, **P < 0.01 ***P < 0.001 and ****P < 0.0001 for treated cells vs control]

We found that 5 mRNAs were significantly upregulated, and 4 mRNAs were significantly downregulated in U87-MG cells that were treated with CDC7 inhibitor. Pathway analysis and the list of differentially expressed genes is shown in Fig. 6a.

In parallel to mRNA expression analysis, we also aimed to identify how CDC7 inhibition alters the cellular miRNA profile. By using a commercial PCR array specific to known miRNAs related to different tumors of the brain and central nervous system, we determined that CDC7 inhibition leads to upregulation of hsa-miR-451a (2.34-fold) and hsa-miR-320a (1.96-fold) in U87-MG cells. To validate the results of our profiling experiment, we used real-time PCR, and found that CDC7 inhibition results in 2.2-fold increase in miR-451a expression in U87-MG cells (Fig. 6e).

We wanted to focus on CTSS, as it was the most significantly downregulated target in the PCR array. To validate our screening results, we analyzed CTSS mRNA and protein levels after CDC7 inhibitor treatment. Treatment with CDC7 inhibitor (2.5 µM) for 48 h resulted in 55% decrease in CTSS mRNA expression in U87-MG cells, and 48% decrease in U251-MG cells (Fig. 6c). Similarly, treatment with CDC7 inhibitor (2.5 µM) for 72 h caused a decrease in CTSS protein expression in U87-MG cells (Fig. 6b, d). Taken together, these results suggest that CDC7 inhibition may suppress glioblastoma growth through CTSS.

## Discussion

Cancer cells override the checkpoints in cell cycle to sustain continuous growth; however, this results in replicative stress. Recently, it has been suggested that replicative stress can be enhanced in a targeted way to attenuate growth of cancer cells. In this regard, CDC7 has been suggested as a potential therapeutic target for p53-negative breast cancers [17], and other cancer types, as it is known to be overexpressed in multiple cancers [6, 18].

Commonly used inhibitors of DNA replication (including topoisomerase inhibitors, intercalating agents, alkylating agents) lead to DNA damage, and activate the S-phase replication checkpoint (Chk1/Chk2-dependent checkpoint) [11]. Contrary to these agents, CDC7 inhibitors are able to halt DNA replication, and induce apoptosis without activating the Chk1-dependent pathway [11]. This brings a therapeutic advantage to this class of agents.

PHA-767491 hydrochloride is a first-generation CDC7 inhibitor with well-characterized anti-tumor activity. In the pioneering study, Montagnoli et al. showed that PHA-767491 hydrochloride inhibits cell proliferation in a comprehensive panel of cancer cell lines at a mean IC50 value of 3.17 µM [12]. The authors also showed that PHA-767491 hydrochloride induces apoptosis in multiple cell lines [12]. In our study, we determined that PHA-767491 effectively reduces cell viability in both glioblastoma cell lines.

The *IDH1* gene encodes isocitrate dehydrogenase 1, which is responsible for catalyzing the conversion of isocitrate to alpha-ketoglutarate. The most common IDH1 mutation is R132H, which produces a missense mutation that affects substrate binding ability of the enzyme. This mutation is commonly found in secondary glioblastomas, and has clinical importance, as it is associated with improved survival in glioblastoma patients [19].

The IDH1 status of experimental cell models is a crucial factor to consider when evaluating the therapeutic potential of CDC7 inhibition. The study by Ichimura et al. showed that none of the 15 glioblastoma cell lines (including U87-MG and U251-MG) has IDH1 mutations [20]. To address whether IDH1 status is associated with a differential response to CDC7 inhibition, further studies should be carried out on IDH1 wild-type and IDH1 mutant cell lines.

The methylation of *MGMT* promoter is another factor that is associated with survival of glioblastoma patients. The *MGMT* gene encodes O^6^-methylguanine–DNA methyltransferase, which functions as a DNA-repair protein to remove alkyl groups from guanine residues. The methylation of MGMT promoter has a direct clinical consequence, as it is associated with longer overall survival in patients with glioblastoma, who undergo a combination treatment consisting of radiotherapy and an alkylating agent (such as temozolomide) [21].

Both U87-MG and U251-MG cell lines have hypermethylated *MGMT* promoter [22, 23]. Considering that CDC7 inhibition with PHA-767491 does not elicit DNA damage response in recipient cells, it is possible that *MGMT* methylation status is not associated with a differential response to CDC7 inhibition. The consequences of CDC7 inhibition should be compared between cell lines that have normal *MGMT* promoter and cell lines with hypermethylated *MGMT* promoter to reach a better conclusion.

To analyze the effects of CDC7 inhibition on non-tumorigenic cells, we used 3T3 cell line as a model system. Our results indicate that CDC7 inhibition leads to a slight decrease in cell viability, while causing a significant reduction in cell proliferation. In addition, CDC7 inhibition does not induce apoptotic cell death in 3T3 cells, unlike glioblastoma cells. While CDC7 inhibition appears to cause selective cytotoxicity in glioblastoma cells, these results should be confirmed further in primary human astrocytes and other non-tumorigenic cells.

To understand the molecular consequences of CDC7 inhibition, we used two commercial PCR arrays, which contain probes against (1) miRNAs known to be associated with brain and central nervous system tumors, and (2) mRNAs with roles in key intracellular signaling pathways. We determined that several important genes show differential expression in response to CDC7 inhibition. Considering that the majority of these genes are classified as growth factors and receptors, it is possible that glioblastoma cells are trying to compensate the tumor suppressive effect of CDC7 inhibition by activating key genes related to cell survival. This finding is particularly important, as it may allow predicting how cells are likely to develop chemoresistance over time. By identifying upregulated gene(s) and associated pathways, it may be possible to design combined therapies for glioblastoma.

hsa-miR-451a was one of the few miRNAs, which shows differential expression in response to CDC7 inhibition. We determined that CDC7 inhibition leads to a 2.3-fold increase in hsa-miR-451a expression in U87-MG cells. Previously, hsa-miR-451/AMPK complex was shown to work as a conditional switch to regulate glioblastoma cell migration and proliferation [24]. In case of glucose deprivation, hsa-miR-451 expression is reduced, which in turn leads to enhanced cell migration and survival [25]. In a recent study, Drusco et al. found that miR-451, among several other miRNAs, is significantly upregulated in cerebrospinal fluid (CSF) sample of glioblastoma patients (>180-fold compared to normal) [26]. These findings are consistent with our results, which indicate that CDC7 inhibition results in upregulation of hsa-miR-451 expression and decreased cell migration.

Cathepsin S is a lysosomal cysteine protease, which is encoded by the *CTSS* gene. CTSS is overexpressed in different cancer types (including astrocytomas [27]), and is reported to be an independent prognostic factor of survival for glioblastoma [28]. Previously, Zhang et al. reported that inhibition of CTSS expression induces autophagy and apoptosis in glioblastoma cell lines, through ROS-mediated PI3K/AKT/mTOR/p70S6K and JNK signaling pathways [29]. In another study, Kenig et al. found a correlation between increased CTSS expression and invasiveness of glioblastoma cells [30]. Recently, Sevenich et al. reported that CTSS expression is significantly high in tumor cells in early stage metastases, and pharmacological inhibition of CTSS reduces experimental brain metastasis [31].

## Conclusion

In this study, we focused on multiple aspects of glioblastoma biology to understand the consequences of CDC7 inhibition in vitro. We provided substantial evidence that CDC7 inhibition suppresses glioblastoma growth and invasiveness. In addition, our results provide preliminary evidence that CDC7 inhibition alters the glioblastoma transcriptome by upregulating and downregulating key genes involved in different cellular pathways. Notably, our results suggest that CDC7 inhibition may suppress glioblastoma growth through CTSS.

## Additional file

**Additional file 1: Figure S1.** The effects of CDC7 inhibition on non-tumorigenic cells. A. 3T3 cells were treated with different concentrations of CDC7 inhibitor (2.5 and 10 μM) for 72 h, and PrestoBlue cell viability reagent (Thermo Fisher Scientific, #A13261) was used to assess cell viability. B. Under similar experimental conditions, a chemiluminescent BrdU incorporation assay (Cell Signaling, #5294) was used to assess the rate of cell proliferation. C. In parallel to cell viability and cell proliferation assays, Cell Death Detection ELISA^Plus^ (Roche, #11544675001) was used to assess apoptotic death. Data represent mean ± SEM. of five biological replicates. [*P < 0.05, **P < 0.01 ***P < 0.001 and ****P < 0.0001 for treated cells vs control].

## Abbreviations

AMPK 5′: adenosine monophosphate-activated protein kinase
BrdU: bromodeoxyuridine
CDC7: cell division cycle 7-related protein kinase
cDNA: complementary deoxyribonucleic acid
CSF: cerebrospinal fluid
CTSS: cathepsin S
DMEM: Dulbecco’s Modified Eagle’s Medium
DNA: deoxyribonucleic acid
FBS: fetal bovine serum
GAPDH: glyceraldehyde 3-phosphate dehydrogenase
IC_50_: half maximal inhibitory concentration
IDH1: isocitrate dehydrogenase 1
JNK: mitogen-activated protein kinase 8
MCM2: minichromosome maintenance protein 2
p-MCM2: phospho-minichromosome maintenance protein 2
MGMT: O^6^-methylguanine-DNA methyltransferase
mRNA: messenger ribonucleic acid
miRNA: micro ribonucleic acid
mTOR: mechanistic target of rapamycin
p53: tumor protein 53
p70s6k: phosphoprotein 70 ribosomal protein S6
PBS: phosphate-buffered saline
PCR: polymerase chain reaction
PI3K: phosphoinositide 3-kinase
ROS: reactive oxygen species
