# Supplementary material for: Cell division cycle 7-kinase inhibitor PHA-767491 hydrochloride suppresses glioblastoma growth and invasiveness
Source: Cancer Cell Int. 2016 Nov 18;16:88. doi: 10.1186/s12935-016-0364-8 (PMC5116134; doi:10.1186/s12935-016-0364-8)

**Supplementary Materials**

**Supplementary Figure 1. The effects of CDC7 inhibition on non-tumorigenic cells.** A. 3T3 cells were treated with different concentrations of CDC7 inhibitor (2.5 and 10 μM) for 72 hours, and PrestoBlue cell viability reagent (Thermo Fisher Scientific, #A13261) was used to assess cell viability. B. Under similar experimental conditions, a chemiluminescent BrdU incorporation assay (Cell Signaling, #5294) was used to assess the rate of cell proliferation. C. In parallel to cell viability and cell proliferation assays, Cell Death Detection ELISA^Plus^ (Roche, #11544675001) was used to assess apoptotic death. Data represent mean ± S.E.M. of five biological replicates. [*P < 0.05, **P < 0.01 ***P < 0.001 and ****P<0.0001 for treated cells vs control].


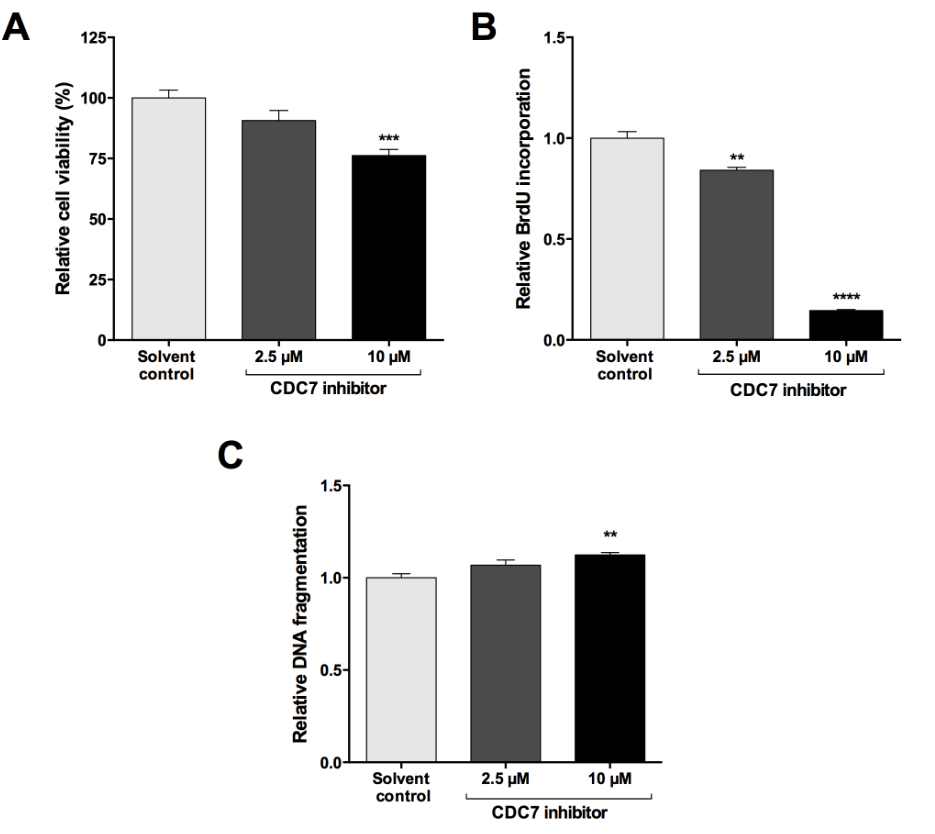

Supplement: Supplementary file 1 — Additional file 1: Figure S1. The effects of CDC7 inhibition on non-tumorigenic cells. A. 3T3 cells were treated with different concentrations of CDC7 inhibitor (2.5 and 10 μM) for 72 h, and PrestoBlue cell viability reagent (Thermo Fisher Scientific, #A13261) was used to assess cell viability. B. Under similar experimental conditions, a chemiluminescent BrdU incorporation assay (Cell Signaling, #5294) was used to assess the rate of cell proliferation. C. In parallel to cell viability and cell proliferation assays, Cell Death Detection ELISAPlus (Roche, #11544675001) was used to assess apoptotic death. Data represent mean ± SEM. of five biological replicates. [*P < 0.05, **P < 0.01 ***P < 0.001 and ****P < 0.0001 for treated cells vs control]. [file 12935_2016_364_MOESM1_ESM.docx]
